# A Novel Fully Human Agonistic Single Chain Fragment Variable Antibody Targeting Death Receptor 5 with Potent Antitumor Activity In Vitro and In Vivo

**DOI:** 10.3390/ijms18102064

**Published:** 2017-09-27

**Authors:** Gaoxin Lei, Menglong Xu, Zhipan Xu, Lili Gu, Chenchen Lu, Zhengli Bai, Yue Wang, Yongbo Zhang, Huajing Hu, Yiwei Jiang, Wenfeng Zhao, Shuhua Tan

**Affiliations:** State Key Laboratory of Natural Medicines, School of Life Science and Technology, China Pharmaceutical University, Nanjing 210009, China; raystar-85@163.com (G.L.); xumenglong_605@163.com (M.X.); zhipanxu@126.com (Z.X.); lyguli2008@163.com (L.G.); luchenchencpu@yeah.net (C.L.); 15211030549@stu.cpu.edu.cn (Z.B.); wangyuejn@163.com (Y.W.); yongbozhome@126.com (Y.Z.); huhuajing_0124@126.com (H.H.); yiweijiang90@163.com (Y.J.); wenfengzhaocpu@126.com (W.Z.)

**Keywords:** death receptor 5 (DR5), human antibody, single chain fragment variable (scFv), apoptosis, phage display

## Abstract

Agonistic antibodies, which bind specifically to death receptor 5 (DR5), can trigger apoptosis in tumor cells through the extrinsic pathway. In this present study, we describe the use of a phage display to isolate a novel fully human agonistic single chain fragment variable (scFv) antibody, which targets DR5. After five rounds of panning a large (1.2 × 10^8^ clones) phage display library on DR5, a total of over 4000 scFv clones were screened by the phage ELISA. After screening for agonism in a cell-viability assay in vitro, a novel DR5-specific scFv antibody TR2-3 was isolated, which inhibited COLO205 and MDA-MB-231 tumor cell growth without any cross-linking agents. The activity of TR2-3 in inducing apoptosis in cancer cells was evaluated by using an Annexin V-PE apoptosis detection kit in combination with flow cytometry and the Hoechst 33342 and propidium iodide double staining analysis. In addition, the activation of caspase-dependent apoptosis was evaluated by Western blot assays. The results indicated that TR2-3 induced robust apoptosis of the COLO205 and MDA-MB-231 cells in a dose-dependent and time-dependent manner, while it remarkably upregulated the cleavage of caspase-3 and caspase-8. Furthermore, TR2-3 suppressed the tumor growth significantly in the xenograft model. Taken together, these data suggest that TR2-3 exhibited potent antitumor activity both in vitro and in vivo. This work provides a novel human antibody, which might be a promising candidate for cancer therapy by targeting DR5.

## 1. Introduction

Apoptosis is a process of programmed cell death event, which eliminates harmful cells from the body in order to maintain tissue integrity [1]. Apoptosis can be triggered through two pathways. The first is the intrinsic pathway, also known as the mitochondrial pathway, which is activated by intracellular signals generated in response to cellular stress and depends on the release of pro-apoptotic factors from the mitochondria. The second is the extrinsic pathway, which is initiated by death receptors belonging to the tumor necrosis factor receptor (TNF) receptor superfamily [2,3,4].

The TNF-related apoptosis-inducing ligand (TRAIL) is a type-II membrane protein, which initiates apoptosis in a wide variety of human cancer cell lines, but not in most normal human cells [5]. TRAIL interacts as a homotrimer with one soluble neutralizing receptor osteoprotegerin and other four transmembrane receptors—death receptor 4 (DR4/TRAIL-R1), death receptor 5 (DR5/TRAIL-R2), decoy receptor 1 (TRAIL-R3), and decoy receptor 2 (TRAIL-R4) [6,7,8]—of which DR4 and DR5 are essential in TRAIL induced-apoptosis. The binding of TRAIL to death receptors is an initial step of TRAIL-induced apoptosis through the extrinsic pathway with the activation of caspases, a family of cysteine proteases. Thus, recombinant soluble TRAIL has been developed as an anticancer drug in several preclinical and phase I/II studies [6,7,9,10]. However, some limitations have been observed. For example, the half-life of TRAIL is less than 1 h in vivo [11] and the binding of TRAIL to the two decoy receptors could cause drug resistance [8,12]. In theory, the development of agonistic anti-death receptor antibodies with high specificity could avoid the issue of nonspecific binding to decoy receptors and osteoprotegerin, whose serum levels are significantly elevated in a variety of cancers [13,14,15]. Additionally, since the cytotoxic activity of recombinant TRAIL on both hepatocytes and immature erythroid cells has been reported to be predominantly mediated by death receptor 4 [16,17,18], the development of agonistic antibody targeting DR5 might be favorable to address these issues [19,20,21].

The current study aims to isolate a novel fully human DR5 specific agonistic single chain fragment variable (scFv) by constructing and screening a phage library, to assess its apoptosis inducing activity in vitro, and to investigate the therapeutic potential in the xenograft model.

## 2. Results

### 2.1. Preparation of Recombinant Extra-Cellular Domain Sequence of DR5 (sDR5) Protein

**Figure 1:**
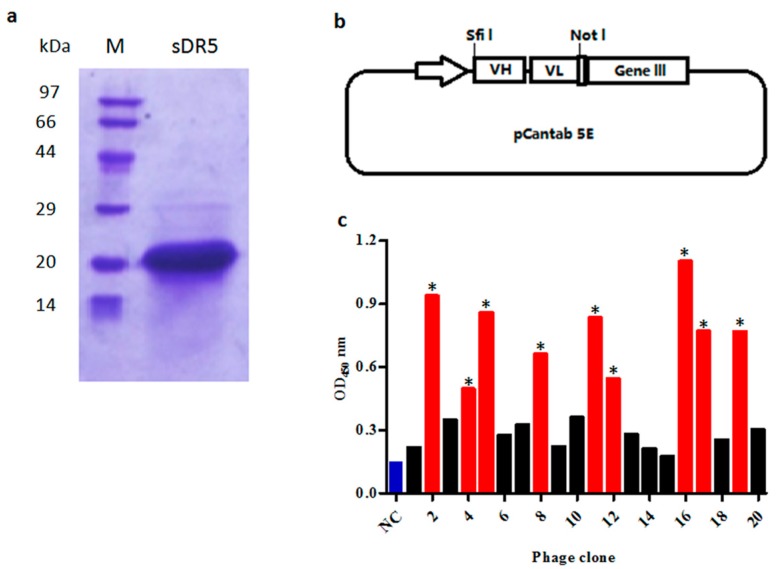
Selection of anti-death receptor 5 (DR5) single chain fragment variable (scFv) antibodies by phage display. (**a**) SDS-PAGE analysis of purified extra-cellular domain sequence of DR5 (sDR5) under reducing conditions; (**b**) The phagemid vector pCANTAB 5E was used for display of scFv antibodies; (**c**) Phage ELISA assay of clones selected on the targeted antigen sDR5. The scFv bound to sDR5 with an ELISA signal at least three-fold greater than the negative control was accepted as being specific (marked *, red bars). Helper phage M13K07 was used as the negative control (NC, blue bar).

To prepare extra-cellular domain sequence of DR5 (sDR5) protein for panning, the 390 bp DNA fragment encoding sDR5 was subcloned into the vector pET21b and expressed in soluble form when induced by isopropyl thiogalactoside (IPTG) in *E. Coli* BL21 (DE3) cells. After being purified by metal affinity chromatography and size-exclusion chromatography, the sDR5 protein was characterized by SDS–PAGE and Coomassie Blue staining. The results showed that the sDR5 protein was highly pure (>90%) with an apparent molecular weight of about 16 kDa (Figure 1a).

### 2.2. Construction of Single Chain Fragment Variable (scFv) Phage Display Library

V-genes of heavy chains and light chains from peripheral blood B-lymphocytes were amplified respectively, before being randomly assembled as VH-(G_4_S_1_)_3_ linker-VL scFv architecture (Figure 1b) by overlapping extension PCR. In total, 72 μg of VH-Vk scFv antibody and 48 μg of VH-Vλ scFv antibody repertoire were purified and ligated into the phage display vector pCANTAB 5E. A total 110 independent transformations generated a scFv phage display library with a repertoire size of 1.2 × 10^8^.

### 2.3. Selection of scFv Antibodies Specific for sDR5

The purified recombinant sDR5 protein was used as a target antigen for antibody selection by a fully human scFv phage display library. In total, five rounds of selection were performed on the immobilized sDR5 at varying antigen concentrations and after a certain number of washings. The DR5-specific scFv from each round was evaluated by the phage ELISA against sDR5 protein. ScFv that bound to sDR5 with an ELISA signal at least three-fold greater than a negative control was accepted as being specific (Figure 1c). A total of over 4000 scFv clones were analyzed by phage ELISA and 442 scFv clones were identified as positive clones. After DNA sequencing of all 442 positive clones, 18 scFv clones were identified, which were different by at least one amino acid.

The isolated scFv clones were designated as TR2-1 to TR2-18. To illustrate the diversity of the 18 different scFv, each scFv sequence was aligned to the human germline VH sequence with IMGT databases. The results demonstrated that these scFv genes were varied in terms of their VH germline. Other gene segments, including JH and DH for heavy chains as well as Vk, Jk, Vl, and Jl for light chains, were all highly diverse (Table 1).

### 2.4. Identification of Agonistic scFv to DR5 Using 3-(4,5-Dimethylthiazol-2-yl)-2,5-Diphenyl Tetrazolium Bromide (MTT) Assay

**Figure 2:**
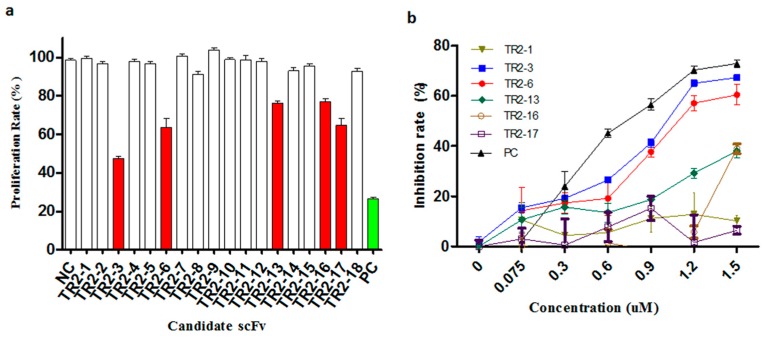
Identification of agonistic anti-DR5 scFv in the cell viability assay. (**a**) Anti-proliferative effect of periplasmic scFv extracts was investigated in a high-throughput 3-(4,5-dimethylthiazol-2-yl)-2,5-diphenyl tetrazolium bromide (MTT) assay on COLO205 cells. Putative agonistic anti-death receptor 5 (DR5) scFv were selected as those that significantly inhibited the COLO205 cells proliferation relative to negative controls (NC). All 18 anti-DR5 scFv were tested and five putative agonistic anti-DR5 scFv antibodies were selected for further study (red bars); The scFv format of Apomab was used as a positive control (PC, green bar). (**b**) After purification of these scFv proteins, the agonistic anti-DR5 scFv was identified by the MTT assay on COLO205 cells. The scFv format of Apomab was included in the same assay as a positive control (PC). These data are representative of at least three independent experiments.

To evaluate the agonistic activity of all selected anti-DR5 scFv antibodies in vitro, the COLO205 cells that are sensitive to TRAIL were used to screen positive clones by 3-(4,5-dimethylthiazol-2-yl)-2,5-diphenyl tetrazolium bromide (MTT) cell viability assay. First, the activity was screened with periplasmic extracts and the five putative DR5 agonistic scFvs were selected by the cell viability assay (Figure 2a). Following this, the five putative DR5 agonistic scFv were transformed into HB2151 cells in order to express the soluble scFv proteins. The obtained yields were in the range of 0.2–3 mg per liter of bacterial culture for the different scFv antibodies. The five scFv antibodies were evaluated more comprehensively in the MTT assay and the two anti-DR5 agonistic scFv antibodies were confirmed (Figure 2b). The most potent anti-DR5 scFv antibody TR2-3 was identified as it inhibited the growth of COLO205 tumor cells with an IC_50_ of 0.9 μM without any cross-linking agents, which has a favorable comparison to the Apomab scFv in the same assay (IC_50_ of 0.6 μM).

### 2.5. Characterization of Isolated TR2-3

**Figure 3:**
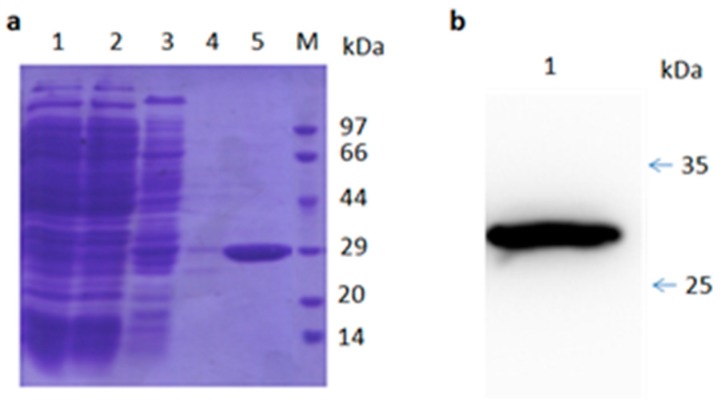
SDS-PAGE and Western blot analysis of purified TR2-3. (**a**) SDS-PAGE analysis of the TR2-3 protein purification by Ni-sepharose column. Lane 1 shows the supernatant of ultrasonic lysed bacterial cell induced by 0.5 mM IPTG overnight; Lane 2 shows flow through; Lane 3 is the elution with 50 mM of imidazole; Lane 4 is the elution with 100 mM imidazole; Lane 5 is the elution with 500 mM of imidazole; and M is the molecular weight marker; (**b**) Western blot analysis of purified TR2-3. Lane 1 shows the purified TR2-3. These data are representative of at least three independent experiments.

Sequencing analysis of TR2-3 revealed that the VH gene belonged to the IGVH3 gene family, in particular, TR2-3 used the VH4-4 gene with a 15-amino acid-long CDR3 sequence (Table 2). On the other hand, the VL gene was derived from the IGVK3-20 family. The VH region of TR2-3 contained 122 amino acids and the VL region contained 108 amino acids. A similarity search by BLASTP revealed that TR2-3 had low homology with known proteins (data not shown), which showed that TR2-3 contained a unique amino acid sequence. SDS–PAGE under reducing conditions showed the TR2-3 band around the theoretical molecular weight of 27.58 kDa (Figure 3).

**Figure 4:**
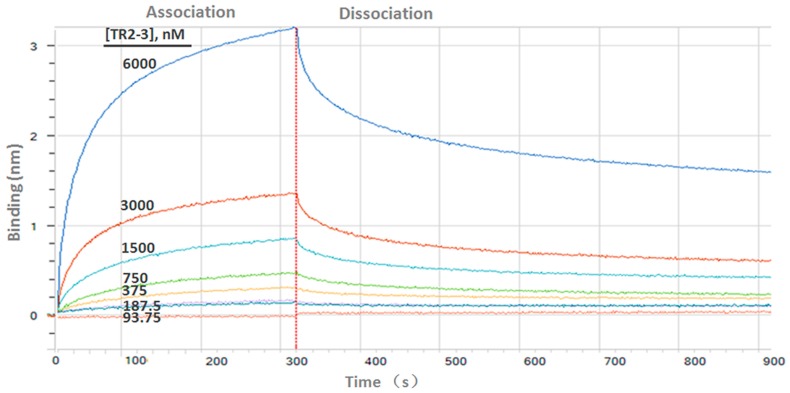
Binding kinetics of TR2-3 to immobilized sDR5 was measured by bio-layer interferometry with the ForteBio Octet QKe System. The determined value of the dissociation constant (KD) was 126 nM (k_on_ 1.15 × 10^4^ M^−1^s^−1^; k_dis_ 1.44 × 10^−3^s^−1^). These data are representative of at least three independent experiments.

**Figure 5:**
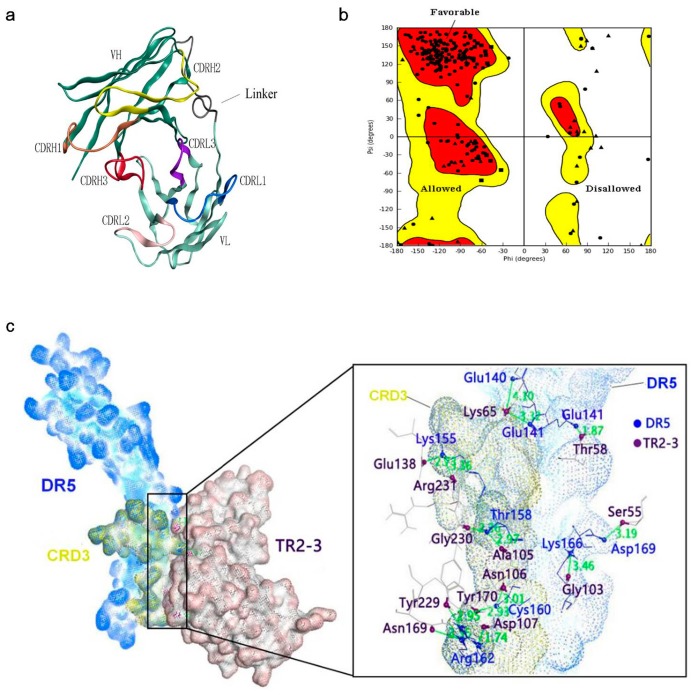
Structural model of the TR2-3 and DR5 complex by homology modeling and molecular dynamics simulations. (**a**) Three-dimensional model of TR2-3 established by homology modeling; (**b**) Ramachandran plot evaluated the reliability of TR2-3 structure; (**c**) The best conformation of TR2-3 and DR5 complex was obtained with the molecular docking analysis and the close-up view of the TR2-3 and DR5 contacts. The residues of DR5 and TR2-3 was colored in blue and violet respectively. These data are representative of at least three independent experiments.

Determination of the kinetic constants of the TR2-3 against immobilized sDR5 demonstrated that the association rate increased with an increase in the concentration of TR2-3 and the kinetic process showed fast association and slow dissociation. According to the calculated results, the value of dissociation constant (KD) was 1.26 × 10^−7^ M or 126 nM (Figure 4). The same method was also used to test the binding affinity of TR2-3 to DR4 or BSA, with the results having showed that there was no cross-reactivity of TR2-3 with DR4 and BSA (data not shown). Moreover, the binding of TR2-3 to DR5 on the cancer cell surface was confirmed by immunofluorescence analysis (Figure S1). Furthermore, the 3D model of TR2-3 (Figure 5a) was established based on the crystal structures of antibody influenza H5 complex (PDB: 4XNM) and transforming growth factor-β neutralizing antibody GC-1008 (PDB: 3EO0) (for similarity score, the values are 89% and 90%, respectively). The reliability of the structure was evaluated by the Ramachandran plot as Figure 5b. Overall, 96.4% (238/247) of all residues were in allowed regions, whereas 3.6% (9/247) of all residues were in disallowed regions. In addition, the structural model passed the evaluation of Verify_3D and 100% of the residues had an average 3D–1D score > 0.2. These data demonstrated that our established structural model of TR2-3 is stereochemically significant and reliable for docking studies. Schrӧdinger Suite 2009 generated 20 combinations of docking poses and the best conformation pose was selected based on the lowest value of binding energies with −54.64 kcal/mol in Prime MM-GBSA panel (Figure 5c). Following this, we performed MOE (molecular operating environment) protein contact against this TR2-3 and DR5 complex, before identifying 12 stable hydrogen bonds and 3 ions bonds between DR5 and TR2-3 (Figure 5c, Table 3). Thus, the hydrogen bond interactions between DR5 and TR2-3 may be an important element for agonistic activity.

**Figure 6:**
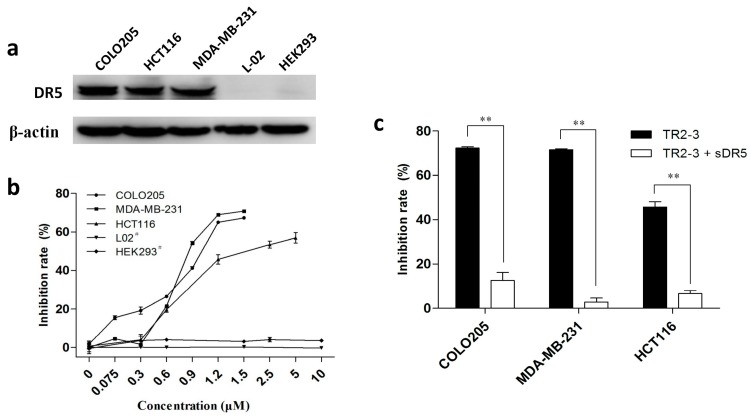
The cytotoxicity of TR2-3 to cancer cells and normal cells. (**a**) DR5 expression level of each cell line was detected by Western blot. Whole-cell lysates from the indicated cell lines were analyzed using antibodies specific to DR5. β-actin was shown as a loading control; (**b**) Cell lines were incubated with TR2-3 at the indicated concentrations for 48 h. # indicates an incubation time of 72 h; (**c**) Cells were incubated with 1.2 μM TR2-3 as well as with 1.2 μM or without soluble DR5. Cell viability was measured by MTT assay. The data represent mean growth inhibition as a percentage of untreated cells in each cell line (** *p* < 0.01, *t*-test). These data are representative of at least three independent experiments.

**Figure 7:**
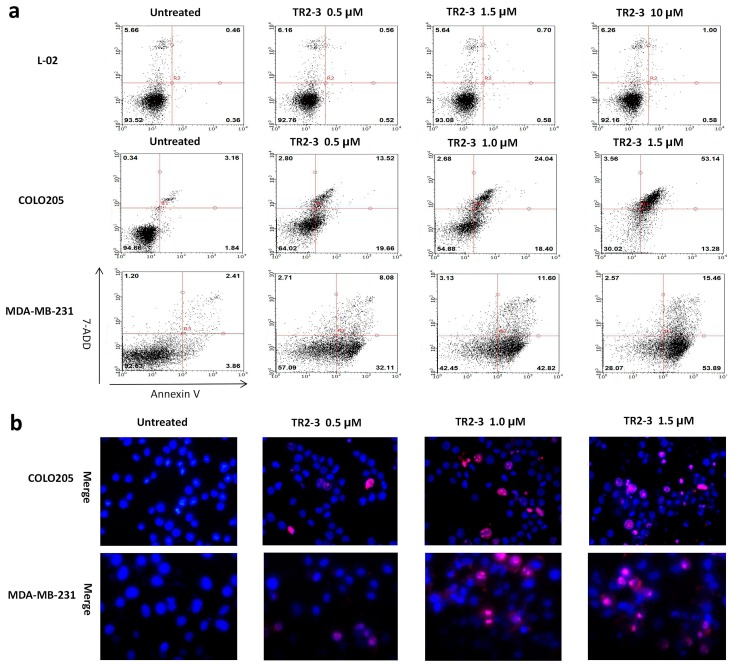
The apoptosis inducing activity of TR2-3 detected in cancer cells and normal cells. COLO205 and MDA-MB-231 cells were treated with 0.5, 1.0, and 1.5 μM or without TR2-3 for 4 h. L-02 cells were treated with a concentration of 1.0, 1.5 and 10 μM or without TR2-3 for 72 h. (**a**) Treated cells were stained by Annexin V-FITC and 7-AAD, counted by flow cytometer (10,000 cells per sample); (**b**) Cells were subjected to Hoechst 33342 (in blue) and propidium iodide (in red) dual staining assays and imaged under a fluorescence microscope (400×). These data are representative of at least three independent experiments.

To investigate the antitumor activity of TR2-3, DR5-positive cancer cell lines—namely COLO205, MDA-MB-231, and HCT116 (Figure 6a)—were treated with TR2-3 at different concentrations for 48 h. All these cells showed significant toxicity over the range of TR2-3 concentrations tested by MTT assay (Figure 6b). In addition, this toxicity can be blocked by adding the same concentration of soluble DR5 (Figure 6c). Moreover, the L-02 and HEK293 cells were used to evaluate the cytotoxicity of TR2-3 to normal cells. The results showed that TR2-3 had no effects on the L-02 and HEK293 cells, as the inhibition rate was not elevated with an increase in the TR2-3 concentration (Figure 6b). Even when the cells were treated with 10 μM of TR2-3 for 72 h, no obvious apoptosis was observed (Figure 7a). These data indicate that TR2-3 exerted no cytotoxicity on normal cells.

**Figure 8:**
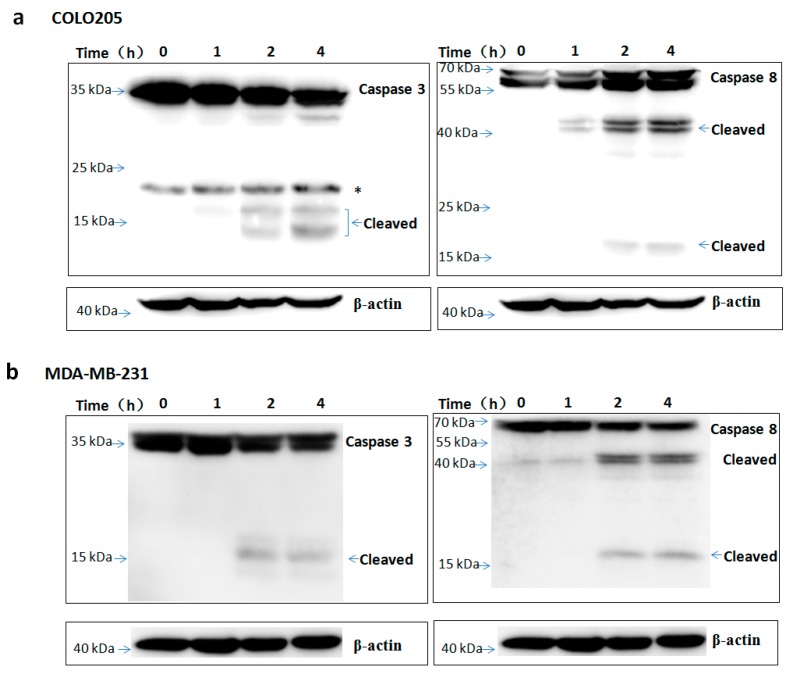
TR2-3 activation of caspase-3 and caspase-8 in COLO205 and MDA-MB-231 cells. The (**a**) COLO205 and (**b**) MDA-MB-231 cells were incubated with TR2-3 for indicated time periods and the whole cell lysates were immunoblotted with the indicated antibodies. * indicates cross-reacting proteins. These data are representative of at least three independent experiments.

To assess the apoptosis inducing activity of TR2-3, the COLO205 and MDA-MB-231 cells were incubated with phosphate buffered saline (PBS) or different concentrations of the TR2-3 proteins for 4 h. Consistent with cell viability results, TR2-3 induced robust apoptosis on the COLO205 cells and MDA-MB-231 in a dose-dependent manner by flow cytometry (Figure 7a) and Hoechst 33342 and propidium iodide double staining analysis (Figure 7b). To confirm these results, caspase-3 and caspase-8 cleavage were tested by Western blots. The results showed that the cleaved caspase-3 and caspase-8 were remarkably upregulated at short incubation times (1–4 h) in the presence of TR2-3 (Figure 8). All these data suggest that TR2-3 exhibited an apoptosis-inducing effect. Activation of caspase-3 and caspase-8 further indicated that TR2-3 induced caspase-dependent apoptosis through the extrinsic pathway.

### 2.6. The TR2-3 Inhibited the Growth of Colon Tumors in Xenograft Model

**Figure 9:**
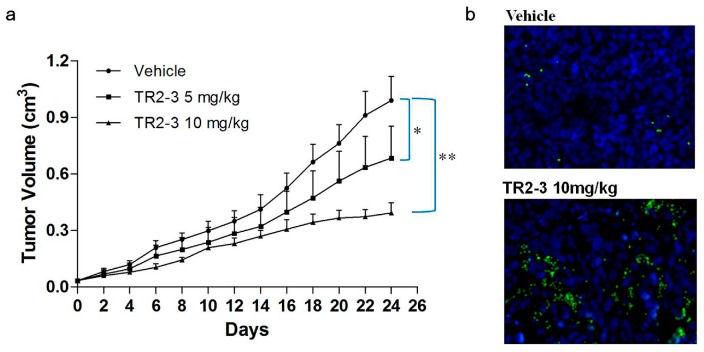
TR2-3 inhibits tumor growth in vivo. COLO205 cells were injected subcutaneously (1 × 10^6^ cells/mouse) to BALB/c nude mice. After eight days, mice were administered PBS as a control or were administered TR2-3 (10 mg/kg) by intraperitoneal injection. (**a**) Each time point represents the mean value of the tumor size on that day as measured by a caliper ruler. Compared with the control, TR2-3 showed a statistically significant higher rate of tumor inhibition (* *p* < 0.05, ** *p* < 0.01 for the *t*-test); (**b**) Representative Immunofluorescence staining of cleaved caspase-3 in sections from the study in (**a**) Cleaved caspase-3 positive cells were visualized in green under a fluorescence microscope (400×) and the cell nuclei were stained with DAPI in blue. These data are representative of at least three independent experiments.

To evaluate the antitumor activity of TR2-3 in vivo, BALB/c nude mice bearing colon (COLO205) tumor xenografts were employed. After three-week treatment with TR2-3 at 10 mg/kg, the COLO205 tumor growth in the xenograft model was significantly inhibited (Figure 9a). As an apoptotic marker, activation of caspase-3 protein was detected in tumor sections by immunofluorescence assay. As expected, activated caspase-3 was almost absent in vehicle slice but prominent in the slice of TR2-3 treatment group (Figure 9b). These results suggest that TR2-3 is a potent antitumor agent for the suppression of tumor growth in vivo.

## 3. Discussion

Antibody-based therapy is one of the most successful and important strategies for oncotherapy [22]. Compared with small molecule drugs, antibodies do not undergo hepatic or renal metabolism. Thus, no marked hepatotoxicity and nephrotoxicity have been found in antibody therapy [23]. The major side effects of antibody therapy relate to their immunogenicity, which may induce injection site reactions, acute hypersensitivity, and other serious complications [23,24]. To avoid immunogenicity issue, we selected fully human anti-DR5 antibodies from the phage display library.

DR5 as a critical component of initiating the extrinsic apoptotic pathway was mainly expressed in cancer cells and rarely in normal cells. Binding of the agonistic antibodies to DR5 could induce the formation of the death-inducing signaling complex (DISC) in cancer cells. DISC activation of initiator caspase-8 induced a cascade of caspase activation where the downstream effector caspase-3 was cleaved and activated, which was followed by the digestion of up to 100 or so proteins causing the cell death [3]. Thus, antibody therapies targeted to DR5 can provide higher specificity and stronger lethality toward cancer cells to improve safety [5,19,25].

In the present study, we utilized recombinant sDR5 protein as target antigens to isolate a novel fully human DR5 specific agonistic scFv antibody TR2-3 by phage display technology. As recent reported [26] and observed in our study, an additional fifth round of panning improved the enrichment efficiency for specific antibodies (data not shown). The in vitro activity study clearly indicated that TR2-3 induced apoptosis in COLO205 and MDA-MB-231 cells as evidenced by the flow cytometric analysis of cell apoptosis and Western blot assays of activated caspase-3 and caspase-8. Similar to other DR5 agonists previously reported [20,21,27], our study showed that TR2-3 induced caspase-dependent apoptosis through the extrinsic pathway. Additionally, TR2-3 showed little effects on normal cells. Taken together, these results indicated that TR2-3 exhibited an apoptosis-inducing effect on tumor cells without causing additional cytotoxicity to normal cells. Furthermore, we evaluate the antitumor activity of TR2-3 in vivo. Treatment with TR2-3 decreased the tumor volume significantly, compared with the vehicle group in the xenograft experiments. The results were in accordance with the preclinical study of DS-8273a, a humanized DR5 agonistic antibody newly generated by Sankyo [28,29], who suggested that TR2-3 is a potent antitumor agent for cancer therapy.

Induction of apoptosis in DR5-positive cancer cells by TR2-3 was significantly blocked when treatment was performed in the presence of the soluble DR5 in all cases. Our observation is supported by a recent report [30] that TR2-3 binding to DR5 at the cell surface of tumor cells was required to induce efficient apoptosis. To evaluate the interaction between TR2-3 and DR5, we detected the binding affinity of TR2-3 to DR5 by bio-layer interferometry with the ForteBio Octet QKe System and established a reliable structural model of the TR2-3 and DR5 complex by homology modeling and molecular dynamic simulations. As expected, TR2-3 showed a high affinity of binding to DR5. In the TR2-3 and DR5 complex structural models, the interaction involves residues from the TR2-3 heavy chain, which complementarily determines the regions (CDR) of H2 and H3 as well as the light chains of CDR L1 and L3. In addition, the heavy chains of CDR H2 and H3 contribute more residues and more surface area than any other region. Furthermore, cysteine-rich domain 3 (CRD3) (residues 75–96) of DR5 had a significant influence as the binding epitope. Consistent with previous research of crystallographic analysis of the Apomab–DR5 Complex [31], the CRD3 domain may be important for DR5 activation.

In summary, we have obtained a novel fully human anti-DR5 scFv antibody TR2-3, which possessed a unique amino acid sequence and specifically induced robust apoptosis of tumor cells in vitro and in vivo. In the future work, we will focus on affinity maturation in vitro using computational design and random mutagenesis targeting CDRs to select the antibodies with higher affinity and higher activity. Another focus will be the expression of a full length human antibody in mammalian cells to study extensively in vivo preclinical tests and human clinical trials in order to investigate their potential utility as human monoclonal antibody therapeutics for cancer therapy. In view of the high efficiency and low toxicity of antibody-based tumor therapy, our research might provide a more effective and safe therapeutic agent to improve the life quality of the patients.

## 4. Materials and Methods

### 4.1. Cell Culture and Reagents

Human colorectal carcinoma cell line COLO205, human colon carcinoma cell line HCT116, human breast cancer cell line MDA-MB-231, human normal liver cell line L-02 and human embryonic kidney cell line HEK293 were obtained from Type Culture Collection of Chinese Academy of Sciences (Shanghai, China). Apart from MDA-MB-231 and HEK293 being maintained in DMEM, other cell lines were maintained in RPMI1640 supplemented with 10% fetal bovine serum.

### 4.2. Cloning, Expression, and Purification of Human Recombinant sDR5 Protein

DR5 cDNA was purchased from Sino Biological INC (Beijing, China). The extra-cellular domain sequence of DR5 (residues 1–130, sDR5) was amplified by PCR and subcloned into pET21b with C-terminal 6× His-tags. The sDR5 protein was expressed in *E. coli* BL21(DE3) strain by addition of 0.2 mM IPTG. After induction at 20 °C for 16 h, the bacterial cells were collected by centrifugation, resuspended in phosphate-buffered saline (PBS) and disrupted by sonication. Following this, the sDR5 protein was purified by metal affinity chromatography on Ni-sepharose (GE Healthcare, Chicago, IL, USA), before Superdex 75 HR 10/30 (10 × 300 mm) size-exclusion chromatography was used (GE Healthcare).

### 4.3. Construction of Human Naive scFv Library

Human scFv library was generated as previously described [32]. Total RNA and mRNA were isolated from peripheral blood B-lymphocytes of 105 healthy human donors and 58 sarcoma patients from Zhongda Hospital, which is affiliated to Southeast University. The project was approved by the medical ethics committee of Zhongda Hospital affiliated to Southeast University (No. 2014ZDIIKY12.0, 17 April 2014) and informed consent was obtained from all donors before the study. Following this, the first-strand cDNA was synthesized using gDNA Eraser buffer in PrimeScript™ RT reagent Kit (TaKaRa, Shiga, Japan). V-genes of human heavy chain and light chain were separately amplified by PCR and assembled as VH-(G_4_S_1_)_3_ linker-VL scFv antibodies. The primer sequences can be found in the Supplementary Table S1. The assembled scFv antibody repertoires were purified, cut with Sfi I and Not I, before being cloned into display vector pCANTAB 5E. The scFv vectors were transformed into *E. Coli* TG1 competent cells by electroporation using 0.1 cm gap cuvettes (Bio-rad, North Harbour, New Zealand) in 110 parallel reactions. The scFv library size was estimated by the standard plate colony counting method.

### 4.4. Selection of DR5 Binding scFv Antibodies from Human Naive scFv Library

#### 4.4.1. Biopanning of the scFv Phage Library

Immunotubes (Nunc, Roskilde, Denmark) were respectively coated with a two-fold serial dilution concentration of 3.75–60 μg/mL sDR5 protein in a carbonate/bicarbonate buffer. Following this, a large human scFv phage display library containing 1.2 × 10^8^ clones was bio-panned against recombinant sDR5 according to the standard methods as described [33]. Detailed description can be found in the supplementary material.

#### 4.4.2. Phage ELISA

After five rounds of panning, the amplified phage of individual TG1 clones were prepared as described [34]. A total of 100 μL of phages was added to sDR5-coated ELISA plates, which was incubated at 37 °C for 2 h. The plates were washed with PBST (0.1% Tween 20 in PBS) and subsequently, a mouse anti-M13 horseradish peroxidase (HRP) conjugate antibody (Sino Biological INC) was added. Followed by an incubation of 2 h at 37 °C and washing, the TMB peroxidase substrate was added and the absorbance was read at 450 nm using a microplate spectrophotometer (Thermo Scientific, Waltham, MA, USA).

#### 4.4.3. DNA Sequencing

DNA sequencing was performed at the Genscript Inc (Nanjing, China). The VH and VL gene families were assessed by screening with the IMGT/V-QUEST tool in the IMGT, the ImMunoGeneTics information system^®^ (http://www.imgt.org/IMGT_vquest/share/textes/).

### 4.5. Selection of DR5 Agonist scFv Antibody by MTT Assay

#### 4.5.1. Preparation of Bacterial Periplasmic Extracts

The selected TG1 clones were replicated into a 2 mL plate with 96 wells, which contained 500 μL of 2× YTAG medium (1% yeast extract, 1.6% tryptone, 0.5% NaCl, 100 μg/mL ampicillin, 0.1% glucose). After incubation at 37 °C for approximately 5 h at 200 rpm, the scFv protein expression was induced by 0.5 mM IPTG for 20 h at 30 °C and 250 rpm. Following this, the bacteria were harvested by centrifugation and suspended in 300 μL of TES buffer (50 mM Tris-HCl, 0.5 mM EDTA, 0.5 M Sucrose, pH 7.4). The plates were left on ice for 1 h. Finally, the scFv-enriched bacterial periplasmic extract was prepared by centrifugation and used for preliminary screening agonist against scFv antibody.

#### 4.5.2. Purification of scFv Proteins

The selected scFv genes were subcloned into the bacterial expression vector pCANTAB 5E with C-terminal 6× His-tags via *Sfi* I and *Not* I restriction sites. After being confirmed by sequencing, the plasmid constructs were transformed into *E. coli* HB2151 strains. The recombinant strains were grown at 37 °C to the OD_600_ of approximately 0.6 in the Luria-Bertani medium with 100 μg/mL ampicillin. Following this, the protein expression was induced by addition of 0.5 mM IPTG. After 20 h induction at 30 °C, cells were harvested by centrifugation, resuspended in PBS and disrupted by sonication. The scFv protein was purified by immobilized metal affinity chromatography (IMAC), followed by a Superdex 75 size-exclusion chromatography using PBS as running buffer in a Biologic (Bio-Rad) as described previously [35]. Protein concentrations were quantified with the micro-BCA kit (Pierce, Thermo Scientific).

#### 4.5.3. Cell Viability Assay

Cell viability was examined with MTT colorimetry as described [36]. In brief, cells were seeded at 1 × 104 cells/well in 96-well plates and cultured overnight. Following this, cells were treated with 200 μL of fresh medium containing bacterial periplasmic extract or various concentrations of purified scFv antibodies for 48 h. Followed by an additional 4 h with 15 μL MTT (5 mg/mL), cells were analyzed using a microplate spectrophotometer (Thermo Scientific).

### 4.6. Flow Cytometric Analysis of Cell Apoptosis

Apoptosis was measured using a Guava Nexin reagent kit (Merck Millipore, Billerica, MA, USA) according to the manufacturer’s instructions. Briefly, after incubation with fresh medium containing different concentrations of TR2-3 for 4 h, cells were collected and suspended in PBS at a density of 5 × 10^5^ cells/mL. Following this, the 100 μL cell suspension was incubated with 100 μL Guava Nexin reagent for 20 min in the dark at room temperature. The stained cells were detected directly using Guava EasyCyteTM Flow Cytometry (Merck Millipore). A total of 10,000 cells were acquired and the percentage of apoptotic cells were analyzed using Guava EasyCyte™.

### 4.7. Hoechst 33342 and Propidium Iodide Dual Staining Assays of Cell Apoptosis

The induction of apoptosis by TR2-3 was evaluated with Hoechst 33342 and propidium iodide dual staining analysis. Briefly, after treatment with different concentrations of TR2-3 for 4 h, the cells were stained with Hoechst 33342 (100 μg/mL) for 30 min at room temperature. After washing with PBS twice, the cells were stained again with propidium iodide (100 μg/mL) for 10 min at room temperature. The cell morphology was observed under a fluorescence microscope.

### 4.8. Western Blot Assays of Caspase Activation

At the end of treatment with 1 μM of TR2-3 for a different number of times, the cells were harvested and lysed on ice with RIPA buffer. Subsequently, the whole cell lysates were collected by centrifugation and subjected to western blot assays as previously described [10] using the following antibodies: rabbit anti-caspase-8 (polyclonal, BBI, Cardiff, UK), rabbit anti-caspase-3 (polyclonal, BBI), rabbit anti-DR5 (polyclonal, NOVUS, New York, NY, USA), rabbit anti-β-actin (polyclonal, BBI), and goat-anti-rabbit IgG HRP conjugated (polyclonal, BBI). Enhanced chemiluminescence (Pierce) was used for detection.

### 4.9. Homology Modeling and Protein Contact Identification

The three-dimensional (3D) structure model of TR2-3 was built and refined by using Prime of Schrӧdinger Suite 2009 as previously described [37]. In brief, a similarity search by BLASTP and an alignment of multiple sequence structures were performed in Protein Data Bank (PDB). The crystal structures of 4XNM and 3EO0 (PDB ID) were selected as the template for homology modeling because of their sufficiently high homology and best query cover. Following this, the structure reliability of the TR2-3 was evaluated by performing Ramachandran plot and Verify_3D [38]. Subsequently, a high resolution 2.2 Å crystal structure of DR5 (PDB ID:1D4V) was chosen to dock with TR2-3 using Schrӧdinger Suite 2009, which generated the top hit conformations to calculate the binding of free energy using the molecular mechanics/generalized born surface area (MM-GBSA) method of Prime and to identify contact between DR5 and TR2-3.

### 4.10. Binding Affinity Measurements

The binding affinity of scFv antibody to sDR5 or DR4 was measured by bio-layer interferometry with the ForteBio Octet QKe System. The scFv proteins were dissolved in SD Buffer (PBS, pH 7.4, 0.02% Tween 20, 0.1% BSA) at two-fold serial dilutions concentration of 93.75–6000 nM. Meanwhile, the biotinylated sDR5 or DR4 (Sino Biologic INC) proteins were loaded onto the streptavidin sensors (ForteBio, Menlo Park, CA, USA) at a concentration of 1 μM. Following this, the sensors were transferred into the scFv protein solution for association for 5 min, which was followed by SD Buffer for 10 min of dissociation. Sensorgrams were obtained at each concentration and plotted to calculate the dissociation constants by the ForteBio Octet QKe analysis software. The value of KD was calculated as the ratio of rate constants *k*_dis_/*k*_on_, where *k*_dis_ represents the dissociation rate and *k*_on_ represents the association rate.

### 4.11. Preliminary Evaluation of TR2-3's Antitumor Activity In Vivo

Female BALB/c nude mice (six weeks old) were obtained from the Comparative Medicine Center of Yangzhou University. The xenograft model was established by subcutaneously injecting COLO205 cells (1 × 10^6^ cells in 0.1 mL PBS) into the right flank. Tumor volumes were monitored every two days using a vernier caliper based on the formula *V* = ½ × length × (width)^2^. Treatment started when the tumors reached approximately 30–50 mm^3^ in size. The nude mice were randomly divided into three groups (five mice each group): group A were treated with injections of sterile PBS as a vehicle control, while group B and C received TR2-3 at a dose of 5 and 10 mg/kg (once every two days). Mice were sacrificed for analysis before the tumor size reached 1000 mm^3^. The excised tumor tissues were fixed in 4% paraformaldehyde and dehydrated by gradient ethanol and xylene, then embedded in paraffin and processed into 5 μm sections. For active caspase-3 staining, the slides were blocked with 10% normal goat serum (0.1% Tween-20 in PBS, pH 7.4) at room temperature for 30 min and incubated overnight at 4 °C with a monoclonal rabbit anti-cleaved caspase-3 antibody (17 kDa) (Abways Technology, Shanghai, China). After washing in PBS, the slices were treated with Alexa Fluor 488 Goat anti-rabbit IGG. Nuclei were counterstained with DAPI (1:500). The presence of fluorescence was observed under a fluorescence microscope. All the experimental procedures were approved by the Animal Ethics Committee of China Pharmaceutical University (No. 201601078, 7 March 2016).

### 4.12. Statistical Analysis

All data were presented as means ± SE and analyzed in GraphPad Prism 5.0 Software (GraphPad Software, La Jolla, CA, USA). The two-sided Student’s *t*-test was used to evaluate statistically significant differences between two groups both in vitro and in vivo experiments after verification of the normal distribution by the Kolmogorov–Smirnov test. The significance was shown as follows: * *p* < 0.05; and ** *p* < 0.01.

## Figures and Tables

**Table 1:** The V-gene segment usage of the isolated human scFv against sDR5 from the human scFv library.

Clone Name	VH			VL	
	VH Genes	DH Genes	JH Genes	VL Genes	JL Genes
TR2-1	VH3-7	DH3-10	JH3	VL1-51	JL1
TR2-2	VH3-11	DH2-2	JH6	VK1-39	JK2
TR2-3	VH4-4	DH6-13	JH3	VK3-20	JK4
TR2-4	VH3-23	DH6-19	JH4	VL1-51	JL1
TR2-5	VH1-8	DH2-15	JH4	VK3-20	JK3
TR2-6	VH3-23	DH6-19	JH4	VL1-51	JL1
TR2-7	VH3-7	DH3-10	JH3	VL1-41	JL1
TR2-8	VH1-69	DH4-23	JH3	VK3-20	JK2
TR2-9	VH5-51	DH3-22	JH5	VK3-20	JK5
TR2-10	VH4-39	DH3-10	JH5	VK2-30	JK2
TR2-11	VH5-51	DH3-3	JH3	VK3-20	JK1
TR2-12	VH1-46	DH3-3	JH3	VL1-51	JL1
TR2-13	VH3-48	DH2-2	JH5	VK2-28	JK1
TR2-14	VH6-1	DH6-13	JH4	VK3-11	JK4
TR2-15	VH6-1	DH3-3	JH4	VK1-39	JK1
TR2-16	VH3-30	DH2-21	JH6	VL2-14	JL3
TR2-17	VH4-4	DH6-19	JH6	VL1-40	JL1
TR2-18	VH5-51	DH6-19	JH4	VK2-30	JK1

The VH and VL gene segment usage were analyzed in the IMGT database (http://www.imgt.org/IMGT_vquest/share/textes/).

**Table 2:** IMGT–VQUEST sequence analysis of TR2-3.

Amino Acid Sequence of TR2-3
QVQLQESGPGLVKPSGTLSLTCAVSGGSISSSNWWSWVRQPPGKGLEWIGEIYHSGSTNYNPSLKSRVTISVDKSKNQFSLKLSSVTAADTAVYYCARGAAAGTANDAFDIWGQGTMVTVSSGGGGSGGGGSGGGGSETTLTQSPGILSLSPGERASLSCRASQSVPHNYLAWYQQKPGQAPRLLIYGASNRATGIPDRFSGSGSETDFTLTVTRLAPEDFAVYYCQQYGRSLTFGGGTKVEIKRHHHHHH

Underline: CDR1, CDR2, CDR3 regions of heavy chain; Double underline: CDR1, CDR2, CDR3 regions of light chain; Red: (G_4_S)_3_ inter-domain linker; Red underline: 6× His tag; CDR: complementary determining region.

**Table 3:** Protein contacts between DR5 and TR2-3.

Number	Type	Chain	Position	Residue	Chain	Position	Residue	CDR
1	HB	DR5	73	GLU141.OE2	TR2-3	58	THR58.OG1	H2
2	HB	DR5	73	GLU141.O	TR2-3	65	LYS65.NZ	H2
3	HB	DR5	87	LYS155.NZ	TR2-3	138	GLU138.OE1	Fr
4	HB	DR5	87	LYS155.NZ	TR2-3	231	ARG231.O	L3
5	HB	DR5	90	THR158.OG1	TR2-3	105	ALA105A.O	H3
6	HB	DR5	90	THR158.OG1	TR2-3	230	GLY230.O	L3
7	HB	DR5	92	CYS160.O	TR2-3	106	ASN106.ND2	H3
8	HB	DR5	92	CYS160.O	TR2-3	170	TYR170.OH	L1
9	HB	DR5	94	ARG162.NE	TR2-3	169	ASN169.OD1	L1
10	HB	DR5	94	ARG162.NH2	TR2-3	229	TYR229.OH	L3
11	HB	DR5	98	LYS166.NZ	TR2-3	103	GLY103.O	H3
12	HB	DR5	101	ASP169.OD2	TR2-3	55	SER55.OG	H2
13	ION	DR5	72	GLU140.OE2	TR2-3	65	LYS65.NZ	H2
14	ION	DR5	87	LYS155.NZ	TR2-3	138	GLU138.OE1	Fr
15	ION	DR5	94	ARG162.NH1	TR2-3	107	ASP107C.OD1	H3

HB: Hydrogen bond; ION: Ionic bond; and Fr: Framework region.

## Supplementary Materials

Supplementary materials can be found at http://www.mdpi.com/1422-0067/18/10/2064/s1.
