# Supplementary material for: A Novel Fully Human Agonistic Single Chain Fragment Variable Antibody Targeting Death Receptor 5 with Potent Antitumor Activity In Vitro and In Vivo
Source: Int J Mol Sci. 2017 Sep 27;18(10):2064. doi: 10.3390/ijms18102064 (PMC5666746; doi:10.3390/ijms18102064)
Supplement: Supplementary file 1 [file ijms-18-02064-s001.pdf]

Supplementary Materials and Methods: A Novel Fully Human Agonistic Single Chain Fragment Variable Antibody Targeting Death Receptor 5 with Potent Antitumor Activity In Vitro and In Vivo

Gaoxin Lei, Menglong Xu, Zhipan Xu, Lili Gu, Chenchen Lu, Zhengli Bai, Yue Wang, Yongbo Zhang, Huajing Hu, Yiwei Jiang, Wenfeng Zhao and Shuhua Tan

Biopanning of the scFv phage library

A large human scFv phage display library containing 1.2×10^8 clones was used for the selection. Immunotubes (Nunc) were coated with 60 ug/mL sDR5 protein in carbonate/bicarbonate buffer. The library stock was grown in log phase, rescued with M13KO7 helper phage (NEB), and amplified overnight in 2×YTAK (2×YT containing 100 ug/mL ampicillin and 50 ug/mL kanamycin) at 37°C. The phage was precipitated with 4% PEG/0.5 M NaCl and resuspended in PBS buffer. Then the tubes were incubated with phage preparation at 37°C for 2h. After incubation, the tubes were washed 10 times with PBST (PBS containing 0.1% Tween 20) and subsequently with PBS. The bound phage was eluted at 37°C for 30 min with 1 mL of freshly prepared 100 mM HCl solution. The eluted phage particles were incubated with 10 mL of log phase TG1 cells at 37°C with shaking at 150 rpm for 1 h. The infected cells were mixed with glycerol at -80°C. For the next round of panning, 8 mL of infected TG1 cell stock was added to 50 mL of 2×YT medium and grown to log phase. The culture was rescued with M13KO7 helper phage, amplified, precipitated, and used for selection, following the procedure described earlier. The panning process was repeated.

Immunofluorescence analysis

At the end of treatment with 1μM TR2-3 for 1 h, cells were fixed with 100% methanol for 5 min, permeabilized using 0.1% Triton X-100 for 10 min, and then blocked with 10% normal goat serum/PBST buffer for 2 h. After co-incubated with mouse anti-DR5 antibody (PE conjugated, R&D systems) and rabbit anti-His tag antibody (FITC conjugated, Miltenyi Biotec) for 2 h, cells were stained with DAPI for 20 min at room temperature. Followed by washing with PBS twice, the cell morphous was observed under a fluorescence microscope.

Table S1. Primers for PCR amplification of human antibody variable genes.

Primers for primary amplifications of VH genes	
HuVH 1aBACK	5′-CAG GTG CAG CTG GTG CAG TCT GG-3′
HuVH 2aBACK	5′-CAG GTC AAC TTA AGG GAG TCT GG-3′
HuVH 3aBACK	5′-GAG GTG CAG CTG GTG GAG TCT GG-3′
HuVH 4aBACK	5′-CAG GTG CAG CTG CAG GAG TCG GG-3′
HuVH 5aBACK	5′-GAG GTG CAG CTG TTG CAG TCT GC-3′
HuVH 6aBACK	5′-CAG GTA CAG CTG CAG CAG TCA GG-3′
HuJHl-2FOR plus	5′-CGCCTCCACC TGA GGA GAC GGT GAC CAG GGT GCC-3′
HuJH3FOR plus	5′-CGCCTCCACC TGA AGA GAC GGT GAC CAT TGT CCC-3′
HuJH4-5FOR plus	5′-CGCCTCCACC TGA GGA GAC GGT GAC CAG GGT TCC-3′
HuJH6FOR plus	5′-CGCCTCCACC TGA GGA GAC GGT GAC CGT GGT CCC-3′
Primers for primary amplifications of Vκ genes	
HuVκ1aBACK plus	5′-TGGCGGATCG GAC ATC CAG ATG ACC CAG TCT CC-3′
HuVκ2aBACK plus	5′-TGGCGGATCG GAT GTT GTG ATG ACT CAG TCT CC-3′
HuVκ3aBACK plus	5′-TGGCGGATCG GAA ATT GTG TTG ACG CAG TCT CC-3′
HuVκ4aBACK plus	5′-TGGCGGATCG GAC ATC GTG ATG ACC CAG TCT CC-3′
HuVκ5aBACK plus	5′-TGGCGGATCG GAA ACG ACA CTC ACG CAG TCT CC-3′
HuVκ6aBACK plus	5′-TGGCGGATCG GAA ATT GTG CTG ACT CAG TCT CC-3′

HuJκ1 FOR	5′-ACG TTT GAT TTC CAC CTT GGT CCC-3′
HuJκ2 FOR	5′-ACG TTT GAT CTC CAG CTT GGT CCC-3′
HuJκ3 FOR	5′-ACG TTT GAT ATC CAC TTT GGT CCC-3′
HuJκ4 FOR	5′-ACG TTT GAT CTC CAC CTT GGT CCC-3′
HuJκ5 FOR	5′-ACG TTT AAT CTC CAG TCG TGT CCC-3′
Primers for primary amplifications of Vλ genes	
Huλ1BACK plus	5′- TGGCGGATCG CAG TCT GTG TTG ACG CAG CCG CC-3′
Huλ2BACK plus	5′- TGGCGGATCG CAG TCT GCC CTG ACT CAG CCT GC-3′
Huλ3aBACK plus	5′- TGGCGGATCG TCC TAT GTG CTG ACT CAG CCA CC-3′
Huλ3bBACK plus	5′- TGGCGGATCG TCT TCT GAG CTG ACT CAG GAC CC-3′
HL4back plus	5′- TGGCGGATCG CAG CCT GTG CTG ACT CAR YC-3′
Huλ5BACK plus	5′- TGGCGGATCG CAG GCT GTG CTC ACT CAG CCG TC-3′
Huλ6BACK plus	5′- TGGCGGATCG AAT TTT ATG CTG ACT CAG CCC CA-3′
HuJλ1FOR	5′-ACC TAG GAC GGT GAC CTT GGT CCC-3′
HuJλ2-3FOR	5′-ACC TAG GAC GGT CAG CTT GGT CCC-3′
HuJλ7FOR	5′-GAG GAC GGT CAG CTG GGT GC-3′
Primers for second amplifications of VH genes	
HuVH1aBACKSfi	5′-GTC CTC GCA ACT GCG GCC CAG CCG GCC ATG GCC CAG GTG CAG CTG GTG CAG TCT GG-3′
HuVH2aBACKSfi	5′-GTC CTC GCA ACT GCG GCC CAG CCG GCC ATG GCC CAG GTC AAC TTA AGG GAG TCT GG-3′
HuVH3aBACKSfi	5′-GTC CTC GCA ACT GCG GCC CAG CCG GCC ATG GCC GAG GTG CAG CTG GTG GAG TCT GG-3′
HuVH4aBACKSfi	5′-GTC CTC GCA ACT GCG GCC CAG CCG GCC ATG GCC CAG GTG CAG CTG CAG GAG TCG GG-3′
HuVH5aBACKSfi	5′-GTC CTC GCA ACT GCG GCC CAG CCG GCC ATG GCC GAG GTG CAG CTG TTG CAG TCT GC-3′
HuVH6aBACKSfi	5′-GTC CTC GCA ACT GCG GCC CAG CCG GCC ATG GCC CAG GTA CAG CTG CAG CAG TCA GG-3′
Linker-r	5′-CGA TCC GCC ACC GCC AGA ACC ACC TCC GCC TGA ACC GCC TCC ACC-3′
Primers for second amplifications of Vκ genes	
HuJκ1FORNot	5′-GAG TCA TTC TCG ACT TGC GGC CGC ACG TTT GAT TTC CAC CTT GGT CCC-3′
HuJκ2FORNot	5′-GAG TCA TTC TCG ACT TGC GGC CGC ACG TTT GAT CTC CAG CTT GGT CCC-3′
HuJκ3FORNot	5′-GAG TCA TTC TCG ACT TGC GGC CGC ACG TTT GAT ATC CAC TTT GGT CCC-3′
HuJκ4FORNot	5′- GAG TCA TTC TCG ACT TGC GGC CGC ACG TTT GAT CTC CAC CTT GGT CCC-3′
HuJκ5FORNot	5′- GAG TCA TTC TCG ACT TGC GGC CGC ACG TTT AAT CTC CAG TCG TGT CCC-3′
Linker-s	5′-GGT GGA GGC GGT TCA GGC GGA GGT GGT TCT GGC GGT GGC GGA TCG-3′
Primers for second amplifications of Vλ genes	
HuJλ1FORNot	5′-GAG TCA TTC TCG ACT TGC GGC CGC ACC TAG GAC GGT GAC CTT GGT CCC-3′
HuJλ2-3FORNot	5′- GAG TCA TTC TCG ACT TGC GGC CGC ACC TAG GAC GGT CAG CTT GGT CCC-3′
HL7FORNot	5′- GAG TCA TTC TCG ACT TGC GGC CGC GAG GAC GGT CAG CTG GGT GC-3′
Linker-s	5′-GGT GGA GGC GGT TCA GGC GGA GGT GGT TCT GGC GGT GGC GGA TCG-3′
Primers for assembling VH-$(G_4S_1)_3$ linker-VL scFv	
SfiI	5′-GTC CTC GCA ACT GCG GCC CAG CCG GCC ATG GCC-3′
NotI	5′-GAG TCA TTC TCG ACT TGC GGC CGC-3′

Double underline: linker; Underline: restriction sites.

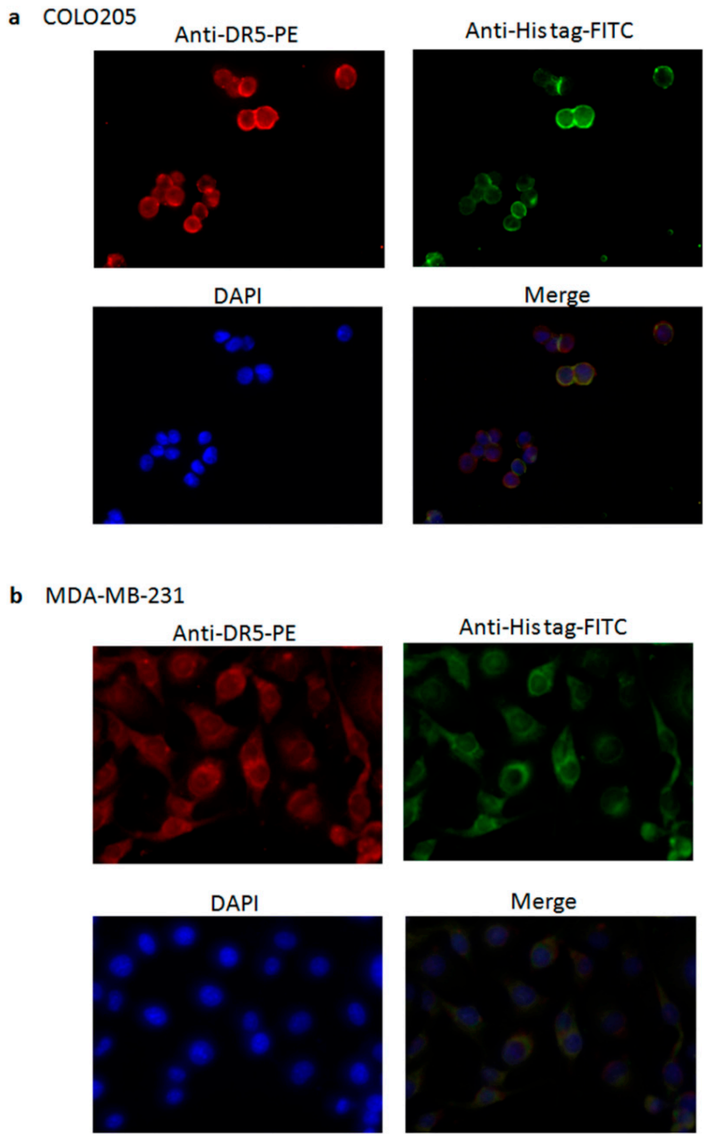

Figure S1. Binding of TR2-3 to DR5 on cancer cell surface was measured by immunofluorescence analysis. At the end of treatment with 1μM TR2-3 for 1 h, COLO205 (**a**) and MDA-MB-231 (**b**) cells were co-incubated with PE conjugated mouse anti-DR5 antibody and FITC conjugated rabbit anti-His tag antibody, and imaged under a fluorescence microscope (400×). Cell nuclei were stained with DAPI in blue.
